# Nipple-Areolar Complex Reconstruction: A Review of the Literature and Introduction of the Rectangle-to-Cube Nipple Flap

**Published:** 2018-03-19

**Authors:** Joshua T. Henderson, Thomas J. Lee, Andrew M. Swiergosz, Andrea R. Hiller, Joshua Choo, Bradon J. Wilhelmi

**Affiliations:** ^a^University of Louisville School of Medicine, Louisville, Ky; ^b^Division of Plastic and Reconstructive Surgery, Department of Surgery, Louisville, Ky

**Keywords:** nipple reconstruction, breast reconstruction, rectangle flap, cube flap, nipple projection

## Abstract

**Objective**: There are many approaches to nipple-areola complex reconstruction. Tissue quality and thickness, desired nipple location and size, scar position, and surgeon preference all play a role in selecting a technique. We present the rectangle-to-cube nipple flap, a new technique for challenging nipple reconstruction. A review of published techniques is compared and contrasted with this flap design. **Methods**: Following bilateral total skin-sparing mastectomies, a patient with breast cancer underwent breast reconstruction with tissue expanders and subsequent nipple reconstruction with the rectangle-to-cube nipple flap. An inferiorly based rectangular flap with medial and lateral extensions is designed inferior to the transverse scar. Upon elevation and rotation, the medial and lateral flaps form a cube. **Results**: In all cases of rectangle-to-cube nipple flaps performed at our institution, adequate nipple projection and patient satisfaction have been achieved at 2-month postoperative evaluation. **Conclusion**: The rectangle-to-cube nipple flap provides sustained nipple projection due to the de-epithelialized base on which the flap sits. The rectangle-to-cube nipple flap also takes advantage of a long transverse scar, and it can be extended to include longer scars for scar revisions.

Nipple-areolar complex reconstruction (NAR), the final stage in breast reconstruction, encompasses numerous surgical techniques. The advantages of each method must be considered to ensure adequate nipple projection and optimal aesthetic result. The rectangle-to-cube nipple flap is a new technique that offers persistent protrusion with the unique advantage of utilization of a long transverse scar.

## METHODS

A 41-year-old woman presented with abnormal screening mammography and was found to have invasive ductal carcinoma of the right breast. The patient elected to have bilateral total skin-sparing mastectomies with immediate breast reconstruction with tissue expanders. 350 CPX3 Mentor tissue expanders were used. After expansion, the expanders were replaced by 650 moderate profile plus Mentor implants filled with 900 mL of saline. She had no postoperative complications, and she desired nipple reconstruction.

Nipple reconstruction was performed 6 months after the permanent implants were placed. Since there was widening of the scar with medial and lateral breast contour redundancy, scar revision was planned concurrently.

## OPERATIVE STEPS

**Figure 1:**
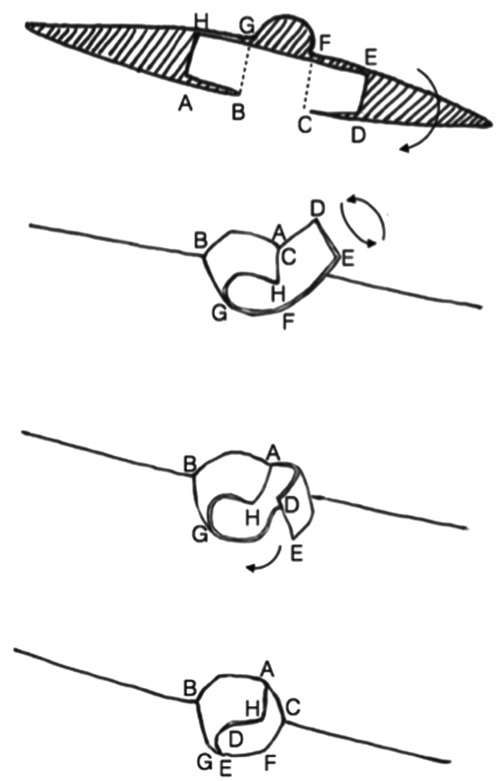
Schematic of flap elevation and rotation. Letters A-D represent the inferior aspect of the flap, whereas letters E-H represent the superior aspect. Letters A and H represent the medial aspect of the flap for NAR of a left breast. The shaded areas are de-epithelialized. An inferiorly based rectangular flap is designed inferior to the transverse scar. The pedicle width is 2 cm, and the length and width of the medial and lateral flaps are both 1.5 cm. The flap is incised full-thickness, and the surrounding scar is de-epithelialized. The medial flap is then elevated and rotated, and point A is secured to the inside corner of the lateral flap and point C with an absorbable suture. The lateral flap is elevated and approximated over the medial flap. Point H of the medial flap is sutured halfway between points C and D of the lateral flap. Points D and E of the lateral flap are then approximated on top of the medial flap using a permanent suture.

**Figure 2:**
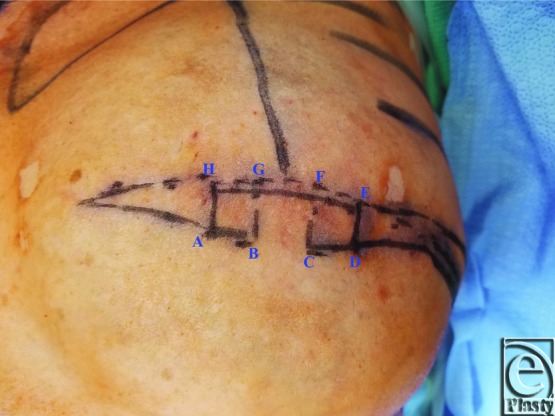
Patient markings.

**Figure 3:**
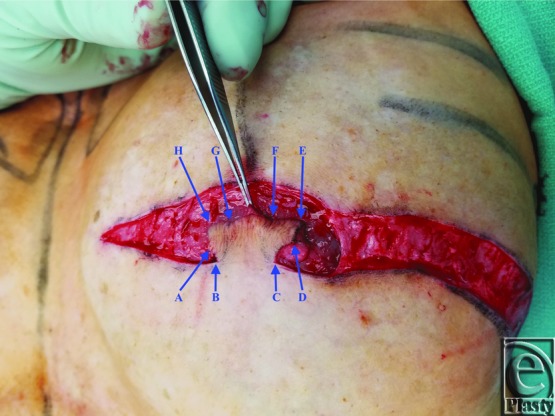
Flap incision and scar de-epithelialization.

**Figure 4:**
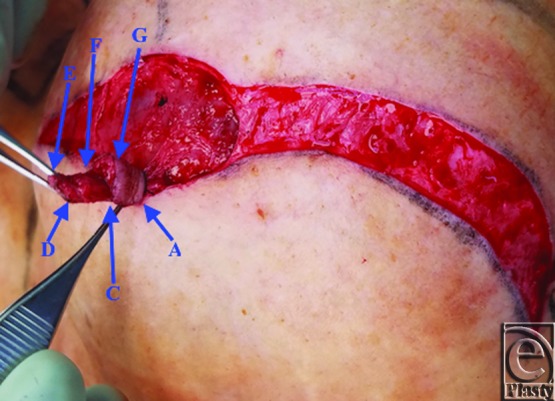
Medial flap rotation.

**Figure 5:**
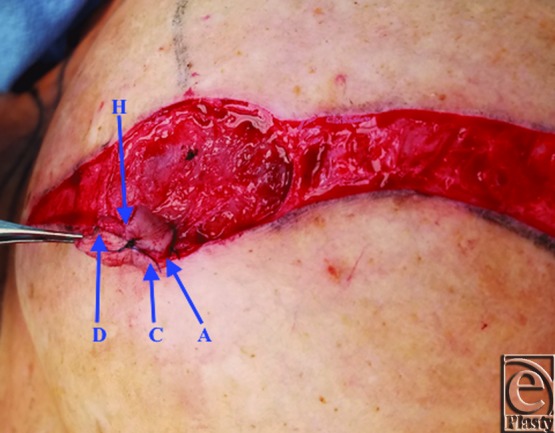
Medial flap inset.

**Figure 6:**
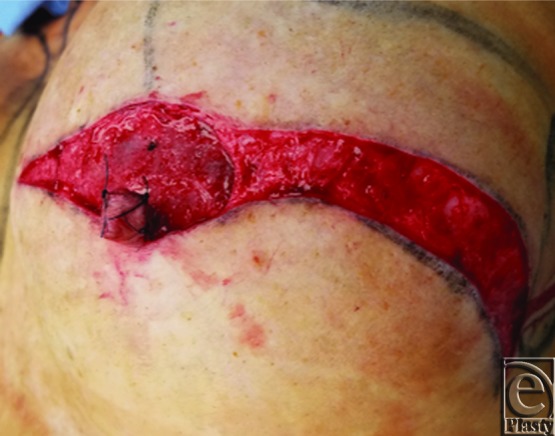
Lateral flap inset.

**Figure 7:**
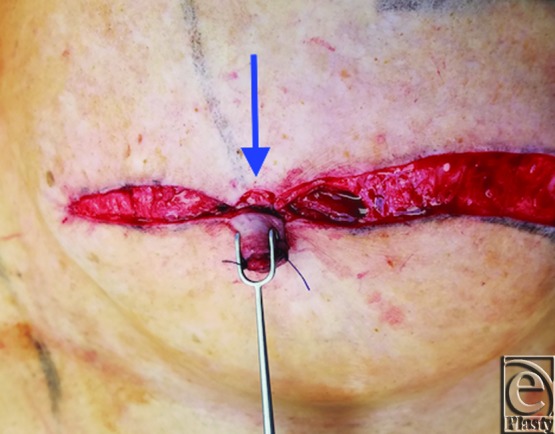
De-epithelialization of half-circle to create a base for the flap.

**Figure 8:**
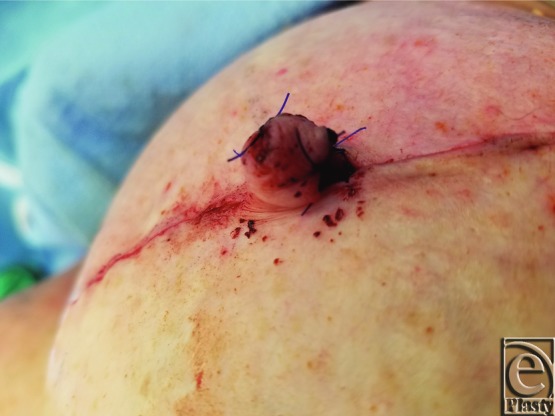
Completed rectangle-to-cube nipple flap.

A schematic for the flap can be seen in Figure 1 with letters corresponding to Figures 2 to 6. Flap markings are made preoperatively. New symmetric breast meridians are approximated using distances from the sternum and mid-clavicle. An inferiorly based rectangular flap is designed inferior to the transverse scar (Fig 2). (A superiorly based rectangular flap can also be designed for a superior pedicle.) The pedicle width is 2 cm, and the length and width of the medial and lateral flaps are both 1.5 cm. The flap is incised full-thickness, and the surrounding scar is de-epithelialized (Fig 3). The medial flap is then elevated and rotated, and point A is secured to the inside corner of the lateral flap, point C, with an absorbable suture (Fig 4). This medial flap creates the base of the reconstructed nipple. The lateral flap is then elevated and approximated over the medial flap. Point H of the medial flap is sutured halfway between points C and D of the lateral flap (Fig 5). Points D and E of the lateral flap are then approximated on top of the medial flap using a permanent suture (Fig 6). A half circle is marked immediately superior to the base of the reconstructed nipple and de-epithelialized to create a stable base upon which the flap can rest (Fig 7). The base of the flap is then sutured to the edge of the de-epithelialized area with a permanent suture (Fig 8).

## RESULTS

**Figure 9:**
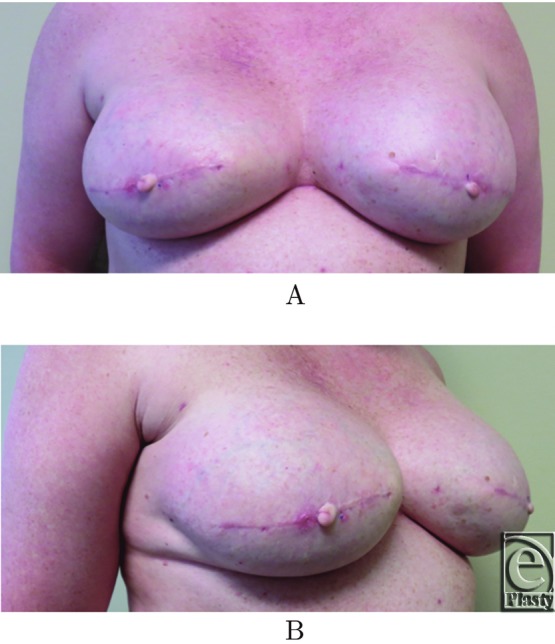
(a,b) Long-term projection.

The patient had no wound-healing issues. Protective dressings were placed over the reconstructed nipples for 4 weeks to allow the rectangle-to-cube nipple flaps to heal. Two-month follow-up showed minimal loss of projection (Figs 9*a* and 9*b*). She is scheduled to return soon for tattooing to complete her reconstruction.

The rectangle-to-cube nipple flap has been performed on 5 patients at our institution who previously had unilateral breast reconstruction, resulting in a transverse scar. Sustained nipple projections were achieved at 3-month postoperative evaluations for each patient.

## DISCUSSION

Breast reconstruction has shown considerable evolution over the past century. In 1895, Vincent Czerny, a professor of surgery at Heidelberg, published a mastectomy case that was reconstructed by transplantation of a fist-sized lipoma from the patient's flank.^1^ Since then, techniques have evolved to reconstruct the aesthetic lost by mastectomy. The final step in breast reconstruction is the creation of the nipple-areola complex. NAR has a psychological contribution to breast reconstruction and has been shown to provide greater satisfaction with regard to sexual behavior and satisfaction with nude appearance.^2^ NAR is often seen as the end of a long and difficult treatment and provides patients a sense of completeness.^3^

Myriad surgical techniques for NAR have been described; however, long-term nipple projection remains a challenge, especially with implant-based reconstructions.^4^^,^^5^ NAR using local tissues is the preferred technique of many surgeons.^5^ The flap options are many and include the skate, star, C-V, double-opposing and V-Y flaps. Table 1 highlights some of the advantages and disadvantages of these flaps and a comparison between them and the rectangle-to-cube flap we present.

Of the numerous methods for NAR, the skate flap design is reported to provide superior nipple projection, capable of maintaining volume more permanently than other techniques.^5^^,^^6^ The skate flap is constructed via a vertical cutaneous fat flap that is elevated with a substantial volume of fatty tissue to offer adequate volume and blood supply to the nipple. Two split-thickness wings are wrapped around the fat core to create a projecting nipple.^7^ The use of this flap is limited in that it is best suited for cases in which a flap-based skin island is present, as is the case with a latissimus dorsi or TRAM (transverse rectus abdominis myocutaneous) flap. It may also be used in cases where the original mastectomy flaps are thick enough to supply sufficient vascularity to the skin from within the reconstructed breast.^8^ The rectangle-to-cube flap is very similar in technique to the skate flap, but it does not require a skin graft.

The star, C-V, and fishtail flaps are additional options that employ wraparound techniques similar to the rectangle-to-cube flap.^9^ With the C-V flap, the advantage of increasing projection by increasing the width of the 2 V flaps is offset by the disadvantage of poor projection in patients with thin skin.^9^ These patients with thin skin sometimes require autologous dermal fat grafting to achieve satisfactory projection.^10^ A shortcoming of the flap arises when a scar exists in the desired location of the NAR, as the C-V flap requires well-vascularized tissue to maintain viability.^9^ Neither the star nor the C-V flap (or its fishtail modification) sits upon a de-epithelialized base. Rather, they are supported mainly by beds of subdermal fat.

Several double-opposing flaps have been proposed and used extensively. The double-opposing tab flap presented by Kroll and Hamilton is a popular technique due to its ease of performance and the ability of its double-opposing flaps to straddle a scar.^11^ It differs from the skate flap by incorporating 2 opposing dermal fat flaps as opposed to only 1 flap. The symmetry of closure of its 2 donor sites also varies considerably from the skate flap and most other flaps.^12^ It is favored in breast reconstruction with a tissue expander or implant because it places the reconstructed nipple at the center of the scar.^13^ Its long-term projection has been reported to be similar to that of the skate flap.^14^ The double-opposing periareolar flap reported by Shestak and Nguyen is unique in its feature of containing all scars from the donor tissue within the peripheral periareolar incision and hiding them easily with intradermal tattooing.^15^ The diamond double-opposing V-Y flap presented by Lesavoy and Liu adds the advantage of its exclusively subcutaneous blood supply avoiding restriction by mastectomy scars.^16^ Incorporating the previous horizontal mastectomy scars into this flap is an obvious benefit; however, the absence of preexisting scars makes this technique less appealing, as it will result in the formation of 2 new scars.^13^^,^^16^ The benefit of including mastectomy scars in the diamond double-opposing V-Y flap is retained in the rectangle-to-cube nipple flap. Our technique allows revision of unattractive mastectomy scars, making it a particularly beneficial technique following skin-sparing mastectomy.

An additional local flap option is the V-Y flap, particularly beneficial in patients with Wise pattern mastectomy scars and in patients requiring revision of a previous nipple reconstruction.^13^ Its incorporation of scar tissue from Wise pattern mastectomy into its reconstruction yields an aesthetically pleasing result.^13^ Its subdermal pedicle decreases retraction forces experienced with most other centrally based flaps, allowing sustained projection.^13^ In addition, the base of the flap can be oriented vertically, allowing incorporation of horizontal scars, but the surgeon must maintain that scars from advancement of the flap likely will not be as well concealed in this orientation.^13^ The V-Y flap shares with the rectangle-to-cube flap the benefit of incorporating a mastectomy scar, but it is best suited for inclusion of a vertical scar. The rectangle-to-cube nipple flap is a much better option for inclusion of a transverse scar.

The rectangle-to-cube nipple flap is a reliable method of NAR for the reconstructive surgeon to add to his or her skill set. It takes advantage of a long transverse scar, and it can be extended to include longer scars for scar revisions. A patient who undergoes a skin-sparing mastectomy that leaves a transverse scar is the perfect candidate for this technique. Although several other techniques utilize medially and laterally extending flaps for reconstruction of the nipple, they do not feature the advantage of extending around the contour of the breast to revise present scars. Importantly, the de-epithelialized halfcircle on which the flap sits aids in maintenance of the nipple projection. The de-epithelialized base provides a stronger foundation of support for the flap than would a bed of subdermal fat. The rectangle-to-cube nipple flap yields an excellent result with sustained projection and should be included in the reconstructive surgeon's armamentarium.

## Figures and Tables

**Table 1:** Comparison of popular flaps with the rectangle-to-cube flap

Technique	Advantages	Disadvantages	Comparison with Rectangle-to-Cube Flap
Skate Flap	Projection	Requires skin graft	No de-epithelialized base
Star Flap	Primary closure	3 visible donor site scars	Similar wraparound technique; no de-epithelialized base
C-V Flap (and Fishtail Flap)	Easy to modify projection by increasing the width of the two V flaps	Poor projection in patients with thin skin, sometimes requiring dermal fat grafting; difficult to place over scar because it requires well-vascularized tissue	Similar wraparound technique; no de-epithelialized base
Double-Opposing Tab Flap	Flaps can straddle a scar	Irregular symmetry of closure	Similar cube configuration; no de-epithelialized base
Double-Opposing Periareolar Flap	Hides donor scars in peripheral periareolar incision	Limited by location of present scars	Donor scars more concealable; no de-epithelialized base
Diamond Double-Opposing V-Y Flap	Exclusive subcutaneous blood supply	Less suitable in the absence of previous mastectomy scars	Incorporates horizontal mastectomy scars; no de-epithelialized base
V-Y Flap	Incorporates scar tissue from Wise pattern mastectomy	Not ideal for breasts with transverse scars	Base of flap can be oriented vertically, allowing incorporation of horizontal scars; no de-epithelialized base
Rectangle-to-cube Cube Flap	Sits upon de-epithelialized base; incorporates transverse scar tissue from skin-sparing mastectomy; can be extended for scar revision	Not ideal for breasts with vertical scars	
